# Lifestyle Intervention in People With Overweight and Obesity and Chronic Low Back Pain: Study Protocol for an International Multicenter Randomized Controlled Trial

**DOI:** 10.1093/ptj/pzaf097

**Published:** 2025-08-13

**Authors:** Melanie Liechti, Alexander P Schurz, Arturo Quiroz Marnef, Jan Taeymans, Ron Clijsen, Heiner Baur, Nathanael Lutz, Tom Deliens, Peter Clarys, Jo Nijs, Matteo Vanroose, Wouter Van Bogaert, Anneleen Malfliet

**Affiliations:** Department of Movement and Sport Sciences, Faculty of Physical Education and Physiotherapy, Vrije Universiteit Brussel, 1050 Brussels, Belgium; School of Health Professions, Bern University of Applied Sciences, 3008 Bern, Switzerland; Department of Movement and Sport Sciences, Faculty of Physical Education and Physiotherapy, Vrije Universiteit Brussel, 1050 Brussels, Belgium; School of Health Professions, Bern University of Applied Sciences, 3008 Bern, Switzerland; Faculty of Medicine, University of Bern, 3012 Bern, Switzerland; Department of Movement and Sport Sciences, Faculty of Physical Education and Physiotherapy, Vrije Universiteit Brussel, 1050 Brussels, Belgium; Pain in Motion Research Group (PAIN), Department of Physiotherapy, Human Physiology and Anatomy, Faculty of Physical Education and Physiotherapy, Vrije Universiteit Brussel, 1090 Brussels, Belgium; Department of Movement and Sport Sciences, Faculty of Physical Education and Physiotherapy, Vrije Universiteit Brussel, 1050 Brussels, Belgium; School of Health Professions, Bern University of Applied Sciences, 3008 Bern, Switzerland; Department of Movement and Sport Sciences, Faculty of Physical Education and Physiotherapy, Vrije Universiteit Brussel, 1050 Brussels, Belgium; School of Health Professions, Bern University of Applied Sciences, 3008 Bern, Switzerland; Rehabilitation and Exercise Science Laboratory RESLab, Department of Business Economics, Health, and Social Care, University of Applied Sciences and Arts of Southern Switzerland, 7302 Landquart, Switzerland; International University of Applied Sciences THIM, 7302 Landquart, Switzerland; School of Health Professions, Bern University of Applied Sciences, 3008 Bern, Switzerland; Department of Movement and Sport Sciences, Faculty of Physical Education and Physiotherapy, Vrije Universiteit Brussel, 1050 Brussels, Belgium; School of Health Professions, Bern University of Applied Sciences, 3008 Bern, Switzerland; Department of Movement and Sport Sciences, Faculty of Physical Education and Physiotherapy, Vrije Universiteit Brussel, 1050 Brussels, Belgium; Department of Physiotherapy, Human Physiology and Anatomy, Faculty of Physical Education and Physiotherapy, Vrije Universiteit Brussel, 1090 Brussels, Belgium; Department of Movement and Sport Sciences, Faculty of Physical Education and Physiotherapy, Vrije Universiteit Brussel, 1050 Brussels, Belgium; Faculty of Medicine, University of Bern, 3012 Bern, Switzerland; Chronic Pain Rehabilitation, Department of Physical Medicine and Physiotherapy, University Hospital Brussels, 1090 Brussels, Belgium; Department of Health and Rehabilitation, Unit of Physiotherapy, Institute of Neuroscience and Physiology, Sahlgrenska Academy, University of Gothenburg, 405 30 Gothenburg, Sweden; Department of Movement and Sport Sciences, Faculty of Physical Education and Physiotherapy, Vrije Universiteit Brussel, 1050 Brussels, Belgium; Pain in Motion Research Group (PAIN), Department of Physiotherapy, Human Physiology and Anatomy, Faculty of Physical Education and Physiotherapy, Vrije Universiteit Brussel, 1090 Brussels, Belgium; Pain in Motion Research Group (PAIN), Department of Physiotherapy, Human Physiology and Anatomy, Faculty of Physical Education and Physiotherapy, Vrije Universiteit Brussel, 1090 Brussels, Belgium; Chronic Pain Rehabilitation, Department of Physical Medicine and Physiotherapy, University Hospital Brussels, 1090 Brussels, Belgium; Pain in Motion International Research Consortium, 1090 Brussels, Belgium; Pain in Motion Research Group (PAIN), Department of Physiotherapy, Human Physiology and Anatomy, Faculty of Physical Education and Physiotherapy, Vrije Universiteit Brussel, 1090 Brussels, Belgium; Pain in Motion International Research Consortium, 1090 Brussels, Belgium; Research Foundation – Flanders (FWO), 1000 Brussels, Belgium

**Keywords:** Body Weights and Measures, Chronic Pain, Exercise Therapy, Low Back Pain, Obesity, Patient Education

## Abstract

**Importance:**

Chronic low back pain (CLBP) is a global health problem with significant clinical, social, and economic challenges. Over 80% of CLBP cases are non-specific (CNLBP), causing the highest number of years lived with disability. People with CNLBP often have comorbidities such as overweight or obesity, which negatively impact symptoms and treatment outcomes.

**Objective:**

The objective is to evaluate whether a lifestyle intervention combining diet, physical activity, and evidence-based physical therapy can reduce pain in individuals with CNLBP and comorbid overweight or obesity.

**Design:**

This is an international multicenter triple-blinded randomized controlled trial (RCT).

**Setting:**

The trial will be conducted in Belgium and Switzerland, with interventions delivered in hospitals (ambulatory care) and outpatient private practices.

**Participants:**

In total, 252 adults will be included and randomly assigned to 1 of 2 treatment arms.

**Interventions:**

The control intervention includes Pain Neuroscience Education and Cognition-Targeted Exercise Therapy. The experimental group receives the same intervention supplemented with a Behavioral Weight Reduction Program.

**Main Outcomes and Measures:**

The primary outcome is pain intensity (assessed using the Brief Pain Inventory). Secondary outcomes include other pain-related outcomes, body composition measures, energy balance related behavior, medical consumption, indirect health-related costs, and quality of life. Assessments will occur at baseline, post-intervention, and at 3-, 6-, 9-, and 12-months follow-up.

**Conclusion:**

This study is the first international multicenter RCT integrating a lifestyle approach into evidence-based physical therapy for people with CNLBP and comorbid overweight or obesity. It will assess whether addressing comorbid overweight or obesity enhances pain reduction and other health outcomes in this population.

**Relevance:**

The results will push the field forward, leading to new knowledge about the (cost-)effectiveness of this approach, which will provide key insights for different stakeholders, help optimizing therapy guidelines and individualized care for people with CNLBP and comorbid overweight or obesity.

## INTRODUCTION

Among musculoskeletal disorders, low back pain (LBP) causes the highest overall burden and years lived with disability globally, resulting in tremendous direct and indirect health care costs (eg, productivity loss).^1^ In about 30% of the LBP cases, pain lasts more than 3 months, and transitions into chronicity (ie, chronic low back pain [CLBP]).^2^ In 80% of cases, CLBP is non-specific in nature (ie, chronic non-specific low back pain [CNLBP]) and is known for its very high incidence and prevalence rates ranging from 3.9% up to 93%.^3^^,^^4^ People with CNLBP often show comorbidities such as overweight and obesity, with different studies showing a close interaction between CNLBP and body composition.^5–8^ Indeed, pain intensity and disability have a dose–response relationship with body mass index (BMI), waist circumference, body fat percentage, and fat mass.^9^ Moreover, overweight and obesity itself are leading risk factors for general disability and increased mortality, resulting in wide-reaching financial consequences.^10–12^

Currently, pain neuroscience education (PNE) and cognition-targeted exercise therapy (CTET) have been shown to be more effective than general education and exercise in managing CNLBP.^13–16^ PNE increases patients’ knowledge of the underlying mechanisms of pain, helping them to reconceptualize pain as less threatening.^17–19^ CTET is based on motor control training and follows a time-contingent approach.^20^ It systematically progresses exercises toward more feared or avoided movements, aiming to break the cycle of pain-related fear, while incorporating therapist-guided discussions to address the patient’s perceptions, concerns, and unhelpful beliefs, thereby reinforcing PNE principles.^13^^,^^15^^,^^20^ Together, PNE and CTET have demonstrated greater reductions in pain and disability, as well as improvements in function in adults with CLBP compared to general exercise and education or exercise alone.^13^^,^^21^^,^^22^

Moreover, overweight and obesity are increasingly recognized as a plausible therapeutic target for people with CNLBP.^23^ Despite their close interaction, few treatment programs for people with CNLBP take overweight or obesity into account and a systematic review identified the need for well-designed, high-quality trials to examine this combined treatment in adults with CLBP and overweight or obesity.^24^^,^^25^ One small-scale proof-of-concept study involving 46 individuals with obesity and CLBP demonstrated that a non-surgical weight loss program, focusing on diet and exercise, not only led to weight loss but also decreased pain (in 48% of participants) and disability (in 70% of participants).^25^ While the findings are compelling, the study’s pre-experimental design limits the ability to draw causal conclusions. Also, in other populations like adults with knee osteoarthritis and overweight or obesity, different high-quality trials showed better outcomes in pain and function when exercise therapy and a dietary change was combined, compared to exercise therapy or dietary changes alone.^26–29^

Given the lack of high-quality studies evaluating the added value of weight management in persons with CNLBP, and the available evidence for its potential in other populations, this article describes the protocol for an international multicenter randomized controlled trial (RCT), which investigates the added value of a lifestyle intervention including a Behavioral Weight Reduction Program (BWRP) to the current best evidence care (ie, PNE and CTET) in people with CNLBP and overweight or obesity. The duration of the intervention for both treatment arms is 14 weeks.

The primary objective of this study is to examine whether adding a BWRP to PNE and CTET compared to PNE and CTET alone, results in less pain in people with CNLBP and overweight or obesity at 12 months follow-up. Secondary objectives focus on the effects in terms of (1) other pain related outcomes, (2) body composition, (3) energy balance related behavior, (4) medical consumption, (5) indirect health costs, and (6) quality of life.

## METHODS

### Study Design

**Figure 1:**
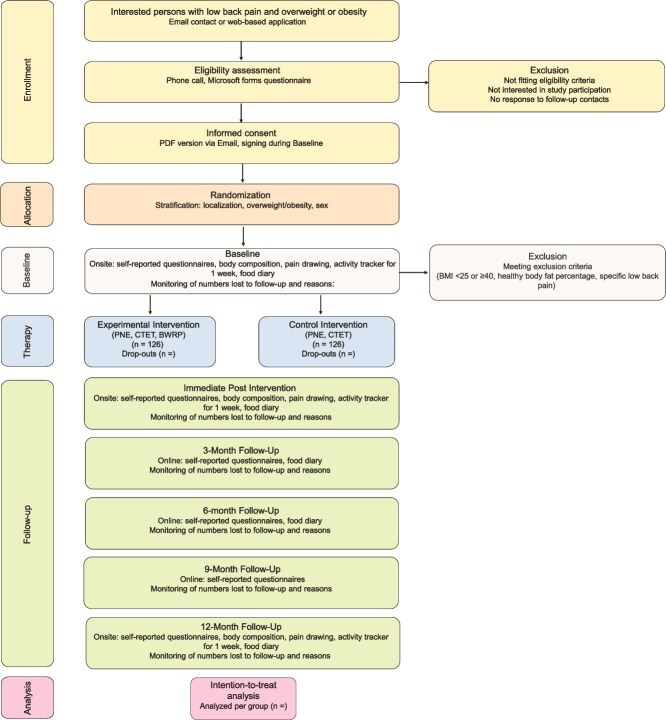
Flow Diagram of the Study. Abbreviations: BMI = Body Mass Index; BWRP = Behavioral Weight Reduction Program; CTET = Cognition-Targeted Exercise Therapy; PNE = Pain Neuroscience Education.

This is the study protocol of the back pain, overweight, obese, weight loss study, a multicenter randomized controlled trial designed to assess the effectiveness of a lifestyle intervention in individuals with overweight or obesity and CNLBP. The international multicenter RCT is conducted in collaboration with the Vrije Universiteit Brussel (Belgium), the Bern University of Applied Sciences (Switzerland), and the University of Applied Sciences and Arts of Southern Switzerland. The protocol follows the Standard Protocol Items: Recommendations for Interventional Trials guidelines^30^ and the Consolidated Standards of Reporting Trials Statement.^31^ The trial was prospectively registered on ClinicalTrials.gov (NCT05811624) in April 2023 and recruitment started in May 2023. To date, 79 patients are included. Figure 1 shows the study flow beginning with an interested participant to the final follow-up assessment.

### Participants

In total, 252 participants with CNLBP and overweight or obesity will be recruited (126 per country). Table 1 depicts a detailed overview of the inclusion and exclusion criteria for participants, study centers, and physical therapists performing the intervention.

**Table 1:** Eligibility Criteria*^a^*

**Criteria for Participants**	
**Inclusion Criteria**	**Exclusion Criteria**
Good knowledge of German/Dutch language (verbal and written)	Severe underlying diseases (eg, diagnosed metabolic disease, diagnosed diabetes, diagnosed cancer, ongoing diagnosed severe depression, diagnosed cognitive impairment, diagnosed eating disorder)
Adults between 18 and 65 years	Being pregnant or given birth in the preceding year, breastfeeding
CNLBP defined according to the NIH Pain Consortium Research Task Force (RTF)^32^: Constant non-specific LBP pain ≥3 months or half of the days in the last 6 months	Specific spinal pathology (eg, disc herniation with clear neuropathic component, spinal stenosis, spondylolisthesis, spinal infection, spinal fracture, malignancy)
Leg pain <7/10 on the NPRS	Dietary or exercise intervention in the last 6 weeks or currently ongoing
BMI overweight ≥25-29.9 kg m^−2^ or obese ≥30-39.9 kg m^−2^^33^	BMI ≥ 40 kg m^−2^
Continuation of usual care 6 weeks prior and during study participation (ie, steady state in usual care)	Healthy body fat %^34^ and healthy WHR (sex and age adjusted)^35^
Signed informed consent	In clarification of invalidity payments
**Criteria for Physical Therapists and Therapy Centers**	
**Inclusion Criteria**	**Exclusion Criteria**
Bachelor of Science in Physical Therapy for Swiss physical therapists and Master of Science in Physical therapy for Belgian physical therapists	Treatment centers only having open treatment rooms, which cannot guarantee privacy and treatment blinding
Fluent English and German or Dutch speaker	
Participation and completion of the trainings provided by the study personnel	
Availability of at least 1 therapist for the experimental and 1 for the control group per treatment location Hospital or private practice setting Treatment rooms that can be closed Similar equipment among the locations/treatment rooms Flexible treatment schedules of physical therapists to guarantee the blinding of participants (eg, no parallel sessions causing participants to see each other in the waiting room)	

^a^ Abbreviations: BMI = Body Mass Index (kg m^−2^); CNLBP = chronic non-specific low back pain; LBP = low back pain; NIH = National Institutes of Health; NPRS = numeric rating scale for pain (0 to 10); WHR = waist-to-hip ratio.

### Recruitment and Informed Consent

Different recruitment strategies will be implemented to reach a diverse group of eligible participants (see Suppl. Material 1). Interested people can apply for study participation on the project website, by directly filling out a first screening questionnaire (Microsoft Forms; Microsoft Corp; Redmond, WA, USA) or by contacting the study team via email or telephone for further information. If the first contact occurs via mail or telephone, the link for the screening questionnaire will be sent afterwards. Participants will be screened for eligibility by research team members, who are not involved in the randomization process or the interventions. All eligible participants will receive an informed consent form (ICF) and are instructed to read it carefully. The ICF will be signed at the beginning of baseline testing, once potential study participants have been given sufficient time to re-read the informed consent and to ask questions. Overweight and obesity were identified based on the Lancet Diabetes & Endocrinology Commission’s recommendation to use a direct body fat measurement or at least 1 anthropometric measurement (eg, waist-to-hip ratio) in addition to BMI.^36^ Therefore, final inclusion will only be confirmed during baseline testing when BMI, body fat percentage, and waist-to-hip ratio are measured on-site. These measurements could lead to exclusion when a participant demonstrates healthy ranges despite having a high BMI, which is possible in athletic individuals and is a known limitation of using BMI as a single parameter to determine overweight or obesity.^36^^,^^37^

### Randomization and Blinding

Patients will be randomly assigned to either the experimental or control group, utilizing a computer-generated number sequence. Randomization is stratified for weight category (overweight/obesity), sex (female/male), country (Belgium/Switzerland), and therapy center. The randomization lists with patient numbers and group allocation will only be accessible by an independent researcher. This researcher coordinates all appointment times with the therapist and assessors in both countries to avoid contact between assessors and therapists, assuring assessors’ blinding. In addition, the participants and the statistician will be blinded for allocation. For participants, this is possible as only a vague description of the therapy is provided in the ICF. Moreover, the therapists providing the experimental treatment are not involved in providing the control intervention and vice versa. The interventions will take place at different times during the day to avoid contamination between groups, and patients will not see each other at the hospital or private practice waiting rooms, nor at the university for measurements. Furthermore, blinding success for both patients and assessors will be evaluated by asking whether they believe the patient received the experimental or control intervention, along with their level of certainty (0% being very unsure, 100% being absolutely sure about the group assignment).

### Settings and Procedures

#### Location Settings

All on-site assessments (baseline, post-intervention, 1-year follow up) will be performed at Vrije Universiteit Brussel, Belgium or Bern University of Applied Sciences, Switzerland. The intervention will be conducted in hospitals (ambulatory care) or private practice rooms of the respective physical therapists in each country.

#### Intervention

Both treatment arms will include 18 sessions spread over a 14-week period, including 17 one-on-one sessions and 1 asynchronous session, where online material will be provided. All treatment sessions will have a duration of approximately 30 minutes and will take place at the cabinet of the respective therapists in Switzerland or in Belgium. If illness or vacation cause absences, participants and therapists are requested to catch up the missed sessions within the 14-weeks’ timeframe if possible. Final number of attended sessions will be recorded. Both treatment arms are organized equally: all participants will receive 3 sessions of PNE in the first 2 weeks, following 15 sessions of CTET (including integration of BWRP for the experimental group) in the remaining 12 weeks. The BWRP intervention will include a lifestyle approach, implying grading daily physical activity and exercise levels alongside of a change in diet to reduce weight. The structure of the sessions and the detailed content of the interventions can be found in Suppl. Material 2 and 3. After the post-intervention measurement, participants will be allowed to receive concomitant care (eg, physical therapy, nutritionist counseling, medication change), which will be monitored with the Medical Consumption Questionnaire during the follow-up period.^38^ Every 3 months, participants will receive and email invitation to complete the follow-up questionnaires via REDCap software (Vanderbilt University, Tennessee, USA) and to fill out a food diary at the 3-month (T2) and 6-month (T3) follow-up (see Table 2). To ensure data completeness, automated reminders will be set up through REDCap, and assessors will contact participants if data remains missing after a reasonable period. After the last follow-up assessment at 12 months (T5) and therefore completion of the study, participants will receive a total remuneration of €120 as travel reimbursement.

**Table 2:** Overview of the Time Schedule of All Assessments*^a^*

**Timepoint**	**Study Period**					
	**Baseline**	**Post-Intervention**	**Follow-Up**			
	**T0**	**T1**	**T2**	**T3**	**T4**	**T5**
**Participant Characteristics**						
**Socio-demographic** Birth year Sex Marital status Occupation Employment status Education Sports (mean duration/week in min) Duration of CNLBP (month) Disease or comorbidities Special diet (eg, vegetarian, vegan, nutrition supplements, food intolerances or allergies)	X					
**Primary Outcome**						
**Pain intensity (BPI)**	X	X	X	X		X
**Secondary Outcome**						
**Pain-related outcomes** Pain distribution (pain drawing)	X	X				X
Pain interference (BPI) Kinesiophobia (TSK) Fear avoidance beliefs (FABQ)	X	X	X	X		X
**Body composition and anthropometrics** Height (SECA stadiometer)	X					
BMI (kg m^−2^) Weight (Tanita) Body fat and fat free mass (TANITA analyzer) Body fat distribution (WC, HC, WHR) Subcutaneous adipose tissue thickness (ultrasound) Muscle thickness (ultrasound)	X	X				X
**Energy balance related behavior** Dietary intake (3-day food diary)	X	X	X	X		X
Sleep quantity (Fitbit Sense) Sleep quality (PSQI)	X	X				X
	X	X	X	X		X
Rest activity cycles (Fitbit Sense) Continuous physical activity (Fitbit Sense)	X	X				X
**Costs** Medical consumption (iMCQ) Indirect health costs (iPCQ)	X	X	X	X	X	X
**Quality of life** Quality of life (EQ-5D-5L)	X	X	X	X	X	X
**Additional Outcomes**						
Therapy adherence		X				
Blinding—group allocation guess						X

^a^ Abbreviations: BMI = body Mass Index; BPI = Brief Pain Inventory; CNLBP = Chronic Non-specific Low Back Pain; EQ-5D-5L = EuroQol 5D-5L questionnaire; FABQ = Fear avoidance belief questionnaire; HC = hip circumference; iMCQ = Medical Consumption Questionnaire; iPCQ = Productivity Cost Questionnaire; PSQI = Pittsburgh Sleep Quality Index; T0 = baseline, 1 week before therapy starts; T1 = post-intervention measurement, 1 week after the 14-week trial; T2 = 3 month follow-up (online); T3 = 6 month follow-up (online); T4 = 9 month follow-up (online), T5 = 12 month follow-up (onsite); TSK = Tampa Scale of Kinesiophobia, WC = waist circumference; WHR = waist-to-hip ratio.

#### Training of Therapists

All sessions will be provided by specially trained physical therapists. The training of PNE and CTET will be provided to all therapists, but only experimental therapists received the BWRP training to avoid treatment contamination. Therapists will not be allowed to switch groups after the training, but the content of the BWRP will be available for the control therapists at the end of the trial. The participants will be treated by the same physical therapist during the whole 14-week period. The training of the therapists will follow standardized intervention manuals (see Supplementary Material 4).

#### Therapy Adherence, Adverse Events, and Discontinuation of the Trial

Therapists will be instructed to document the course of treatment as required by their workplace guidelines. After a participant completes the 14-week therapy period, the therapist will be asked to complete a questionnaire on therapy adherence, compliance, and any adverse events (see Suppl. Material 5). No serious adverse events are expected, and the trial is rated as low-risk category by the ethical committee. Nevertheless, in case of an adverse event, physical therapists will be advised to inform the research team immediately. Furthermore, discontinuation of the trial is considered unlikely. However, if a female participant becomes pregnant or a severe adverse event occurs, participation will be discontinued. In both countries, the principal investigators must inform the respective ethics committee about any adverse events as quickly as possible or within a timeframe of 7 days. Additionally, an annual report on safety, adverse events, and the general progression of the clinical trial will be submitted to the respective ethics committee.

#### Treatment Fidelity

To verify treatment fidelity, therapy sessions will be audio-recorded (only after written consent of the participant) and scored according to pre-defined fidelity criteria (see Suppl. Material 6). These fidelity criteria were developed by the study consortium based on international guidelines and the Cognitive Therapy Adherence and Competence Scale.^39–41^ With this template, the quality of treatment delivery and the therapist’s adherence and competence in delivering the treatment will be scored. Furthermore, the fidelity checklist will be used during training discussions to establish therapists’ readiness, such as their ability to design and implement a gradual exercise program for a hypothetical patient with CNLBP. Moreover, these fidelity criteria will be used during the yearly refresher course for the therapists. In addition, standardized training of therapists based on the treatment manuals will be performed in both treatment arms. Details on the treatment manuals for both treatment arms and the therapists’ training can be found in Supplementary Materials 2–4.

### Outcome Assessment and Data Collection

#### Primary Outcomes

Pain intensity will be measured using the Brief Pain Inventory (BPI) item: “Please rate your pain by circling the number that best describes your pain of the last 24 hours on average (0-10)” will serve as primary outcome.^42^ The primary endpoint is the between-group change in pain intensity from baseline to 12 months follow-up. The BPI is widely used to assess clinical pain and recommended for effectiveness trials in chronic pain^43^ and its reliability and validity has been established.^44^^,^^45^ A reduction of 30% in average pain intensity is considered clinically relevant.^43^ The BPI will be collected online using REDCap at baseline (T0), directly after the intervention (T1, immediate effects), and at 3-months (T2), 6-months (T3), and 12-months (T5) follow-up to capture short-, mid-, and long-term effects, respectively.

#### Secondary Outcomes

Secondary outcome variables included in this study will be other pain related outcomes, different body composition measurements, energy balance-related behavior, medical consumption, indirect health costs, and quality of life. Table 2 shows the timeline of the collection of all outcomes and Table 3 displays the entire list of all outcomes and a detailed description of their assessment.

**Table 3:** Overview and Description of Primary and Secondary Outcome Measures*^a^*

**Construct**		**Measure**	**Description**
**Primary Outcome**			
Pain intensity		Brief Pain Inventory-short form (BPI)	Assessed with 1 question of the BPI: “Please rate your pain by crossing the number that best describes your pain of the last 24 hours on the AVERAGE (0-10).”^42^ The BPI is assessed using the REDCap software.
**Secondary Outcomes**			
Pain-related outcomes			
	Pain interference	Brief Pain Inventory-short form (BPI)	Pain interference is used to assess how much pain interferes with 7 daily activities. It is typically scored as a mean of the 7 items on a 11-point scale (range 0–10), when at least 4 questions have been answered.^42^ The BPI is assessed using the REDCap software.
	Pain extent	Pain drawings on a digital tablet	The Navigate Pain software is used to create pain drawings on a digital tablet (Navigate Pain, Aglance Solutions AALBORG, Denmark). This software enables detailed drawings of pain intensity and the area of discomfort on a 3-D body chart. Analyses can be performed for anatomical and neuroanatomical subdivisions, visualizing pain, and quality of pain with heat maps and can detect map locations, distribution, and patterns of pain and discomfort. This method is feasible and generates reliable and valid data.^46–48^
	Kinesiophobia	Tampa Scale for Kinesiophobia (TSK)	The TSK contains questions about pain-related fear of movement, fear of (re-)injury, and fear avoidance behavior and is frequently used in samples of patients with LBP.^49–51^ A 2-factor model is recommended for patients with CLBP, resulting in 2 subscales: somatic focus (belief of underlying medical problem) and activity avoidance (belief that activity may result in pain or [re]injury).^52^^,^^53^ The TSK is assessed using the REDCap software.
	Fear avoidance beliefs	Fear Avoidance Belief Questionnaire (FABQ)	The FABQ focuses on the beliefs of patients with LBP about how physical activity and work affect LBP (fear of pain caused by physical activity).^54^ Psychometric properties of the German version with a 3-factor model was found to be fair to good, with an internal consistency of 0.91 for the total scale and a good test–re-test reliability of all items (*r* = .87).^54^ The FABQ is assessed using the REDCap software.
Body composition			
	Height	Body height	Height is measured in cm using a stadiometer (SECA 206/213, Hamburg, Germany).
	Weight Body Mass Index (BMI) Body fat and fat free mass	Bioimpedance analysis (BIA)	Bio-impedance analysis is a convenient, low-cost, non-invasive, and reliable tool to assess body composition.^55^ The TANITA Bio-electrical Impedance Analyzer (TANITA MC-780SMA, TANITA Europe B.V., Amsterdam, Netherlands) is used to measure body weight, body fat percentage, and fat free mass (%). Body height and weight will be used to calculate BMI (kg m^−2^). More details can be found in Suppl. Material 7.
	Body fat distribution	Cescorf measuring tape (waist and hip circumference)	Cescorf measuring tape (CESCORF, Porto Alegre/RS, Brazil) is used to assess waist- and hip circumference and to calculate the waist-to-hip ratio, to account for body fat distribution. The body fat distribution should be considered for analyzing the obesity-CLBP relationship.^56^ More details can be found in Suppl. Material 7.
	Adipose tissue thickness Muscle thickness	Uncompressed subcutaneous adipose tissue thickness using a portable ultrasound scanner Muscle thickness of *Musculus multifidus* using a portable ultrasound scanner	To account for regional body composition measurements, uncompressed subcutaneous adipose tissue thickness of triceps, biceps, subscapular, supraspinal, abdominal, and medial calf is assessed using a portable ultrasound scanner (MicrUs Ultrasound scanner TELEMED Ltd, Vilnius, Lithuania), which generates highly reliable and valid data.^57–59^ More details can be found in Suppl. Material 7. In addition, muscle thickness of the *M. multifidus* is assessed using the same ultrasound device.^60–62^ More details can be found in Suppl. Material 7.
Energy balance related behavior			
	Dietary intake	Prospective food diary for 3 day	Dietary intake will be assessed with a 3-day prospective food diary (see Suppl. Material 8). Dietary intake is monitored on 2 representative weekdays and 1 weekend day which can be selected by the participants. The dietary intake includes all food and all drinks/fluids that participants consume. They are advised to weigh their meals and snacks whenever possible and otherwise to describe their portion sizes as detailed as possible (eg, 1 tablespoon of honey). The food diary is sent via WeTransfer service (WeTransfer B.V. Amsterdam, Netherlands) to the study personnel.
	Rest activity cycles	Fitbit Sense (wrist-worn activity monitor)	To capture continuous rest/activity cycles an accelerometry (Fitbit Sense 1 or 2, Fitbit International Limited, Dublin 2, Ireland) is used. This wrist-worn package will be installed 1 week prior to the treatment, will continue throughout the 14 weeks treatment, and will end the day of the follow-up body composition measurement (T1). Participants are instructed to wear the device constantly (day and night) and to synchronize the watch with the app once a day to ensure data storage. At T5, the last follow-up measurement, the Fitbit is handed out again and the participants are instructed to wear the accelerometer for 1 additional week.
	Sleep quality	Pittsburgh Sleep Quality Index (PSQI), Fitbit	The PSQI is a widely used tool to assess sleep quality.^63^^,^^64^ The PSQI is assessed using the REDCap software.
	Sleep quantity	Fitbit Sense	Sleep quantity and quality will be monitored using the Fitbit watch, including sleep schedules, time spent in bed, sleep duration, wake duration, and number of awakenings. While these devices are not recommended for diagnosing sleep disorders, they are suitable for monitoring sleep–wake behavior over extended periods.
	Continuous physical activity	Fitbit Sense	Continuous physical activity and rest/activity cycles are measured with an activity monitor (Fitbit Sense 1 or 2). Data analysis occurs through Fitabase software. Next to physical activity, sedentary behavior will be investigated with the accelerometer data.
Costs			
	Medical consumption	Medical Consumption Questionnaire (MCQ)	Medical consumption (including co-interventions) will be recorded using the Medical Consumption Questionnaire, which is a generic instrument for measuring patients’ total (in-)direct medical consumption.^38^ The MCQ is assessed using the REDCap software.
	Indirect health costs	Productivity Cost Questionnaire (PCQ)	The Productivity Cost Questionnaire will be used to obtain data regarding the indirect costs (eg, costs due to absenteeism or presenteeism).^65^ Costs will be reported in non-aggregated form and will be calculated as “units consumed × unit cost.” Unit costs will be extracted from country specific databases for health care services and drugs. All costs will be expressed in 2026 Belgium euro and for Swiss data also in 2026 Swiss Francs. Intangible costs (eg, pain, stigma) will be omitted. The PCQ is assessed using the REDCap software.
Quality of life			
	Quality of life	EuroQol EQ-5D-5L questionnaire	The EuroQol EQ-5D-5L questionnaire is used to assess quality of life and health profiles, which will be transformed to utilities using Belgium, UK, and German tariffs.^66^^,^^67^ Health utilities are then used to calculate quality adjusted life years for the cost-utility analysis. The EQ-5D-5L is assessed using the REDCap software.
**Additional Outcomes**			
Blinding—group allocation guess		Self-developed questionnaire: Do you think you received the therapy of the experimental or control group?	To measure the level of blinding at the end of the trial, participants answer 1 question for group allocation. They are asked in which group they think they were and how certain they are about this judgement on a scale from 0% to 100% (0% means they are very unsure vs 100%, which means a 100% certainty). This question is assessed using the REDCap software.

^a^ For Swiss participants, questionnaires in German will be used and for Belgian participants, questionnaires in Dutch will be used, respectively. Abbreviations: CLBP = chronic low back pain; LBP = low back pain; UK = United Kingdom.

### Statistical Analysis

#### Sample Size Calculation

A priori sample size calculation for the primary outcome (ie, pain intensity) was performed in GPower version 3.1.9.2 showed that a total sample size of 252 participants (126 in each country) is needed to achieve 95% power to detect a small effect size (partial *η*^2^ = 0.02), using a linear mixed model with alpha level .05 considering a 25% drop-out rate across 12 months of follow-up. Although the effect sizes for the primary outcome measure pain intensity in the proof-of-concept studies were medium to large^13^^,^^25–27^^,^^29^ we anticipate a smaller effect size in the present research proposal (partial *η*^2^ around 0.02), considering the fact that the control intervention has already been shown to be effective in reducing pain.^13^ Taking into account the drop-out rates in the proof of concept studies (9% to 23%),^13^^,^^26^^,^^27^ and the fact that the current proposal combines a weight reduction program with education plus exercise therapy, we anticipate a relatively higher drop-out rate of 25%. Allocation ratio (N2/N1) was defined as 1, resulting in 63 patients in each treatment group per country. A previous trial investigating the effectiveness of a healthy lifestyle intervention for patients with CLBP reported a sample size calculation of 80 participants, accounting for 15% loss to follow-up.^68^ This sample size provided 90% power to detect a clinical meaningful difference in pain intensity of 1.5 points on the Numeric Pain Rating Scale as primary outcome and 80% power to detect a 6% reduction in body weight as a secondary outcome. Based on the small effect size (partial *η*^2^ = 0.02), statistical analyses of other secondary outcome measures in this study should be adequately powered with a sample size of 252 participants.

#### Statistical Analysis Plan

Prior to the analyses, visual inspection for assessing data plausibility will be performed, while the normality assumption will be checked using Q-Q-plots**.** Missing values will be checked for randomness by performing a logistic regression analysis, modeling the probability of missingness against baseline characteristics. If no significant associations are found, missingness will be assumed to be at random**.** Independent samples *t*-tests (for continuous variables) and chi-square tests (for categorical variables) will be performed at baseline to check whether the randomization was successful. If not, variables for which this was not the case will be included as confounders in the main analyses after the direction of the relationship was established using directed acyclic graphs (DAGs).^69^ Drop-out analysis will be performed using independent samples *t*-tests and chi-square tests on the baseline data, while the amount of and reasons for drop-out (participants are asked to give their reasons of withdrawal) will be compared between the experimental and control condition. This procedure will allow us to check whether differential drop-out (and thus attrition bias) is present. Again, variables subject to selection bias will be included as confounders (when identified as such by the DAGs) in the main analyses. General(ized) linear mixed modelling will be performed in R (RStudio version 1.1.383) to evaluate and compare (long-term) therapy effects between the experimental and control intervention. Both the distribution of the outcome measure and residual distributions will be checked for normality using Q-Q-plots. To account for clustering effects (ie, participants will be measured repeatedly, clustered within different hospitals or therapeutic settings), a multilevel model will be constructed, including three levels: (1) repeated measures, (2) clustered within the individual, and (3) clustered within a hospital or other therapeutic setting. This technique is particularly well fitted to analyze repeated measures over time in a hierarchical structure including missing values.^70^ To minimize issues related to multiple testing, contrast testing within the generalized linear mixed model framework will be applied. A priori defined contrasts between the groups will be tested using the R package “multcomp” and the function “glht.” Statistically significant differences will be defined at alpha level .05, and the effect size as well as clinically significant differences will be determined. In addition, numbers needed to treat will be calculated, after transforming the outcome to a binary variable, in analogy with previous research.^71^ Analyses will be performed based on the intention-to-treat principle. Afterward, per protocol analyses will be performed (as a form of sensitivity analysis) to evaluate the effect of therapy adherence and compliance.

For the health economic evaluation, cost-effectiveness and cost-utility planes will be created to illustrate the incremental values of costs and clinical outcomes including the results of a probabilistic sensitivity analysis. If neither of the 2 treatment groups is dominant, incremental cost-effectiveness ratios and incremental cost-utility ratios will be calculated. The analysis will be conducted separately per country and from both health care and societal perspective with a time horizon of 66 weeks.

### Ethics and Dissemination

#### Ethics

This research project will be conducted in accordance with the protocol, the Declaration of Helsinki,^72^ the principles of Good Clinical Practice,^73^ the Human Research Act,^74^ and the Human Research Ordinance,^75^ as well as other locally relevant regulations. Ethical approval has been received from the ethical committees at both study sites: in Switzerland (Swiss Ethics Committee Bern, 2022-02210) and Belgium (Ethics Committee of the University Hospital Brussels BUN: 1432022000296 and the hospital Geel, 2022-394). Both study sites will submit annual safety reports to the respective ethical committee and audit activities (eg, on-demand reviews of the REDCap setup) may be conducted without prior notification to ensure compliance with regulatory standards.

Trial and participant data will be handled with uttermost discretion and will be only accessible to authorized personnel who require the data to fulfil their duties within the scope of the study. On study specific documents, participants will only be identified by a unique participant number. The data files from this study will be managed, processed, and stored in a secure environment (eg, lockable computer systems with passwords, firewall system in place) and by controlling access to digital files with encryption and/or password protection. The coded data will be saved on the network drive of Vrije Universiteit Brussel (the university’s secure environment for private data).

#### Funding

This project is funded by the cross-European Weave program.^76^ As such the Belgian part of the study is funded by the Research Foundation Flanders (reference no. G0894322N), while the Swiss part is funded by the Swiss National Science Foundation (SNF) (reference no. 204659). The funding agencies had no role in the design of the study and will not have any influence in the data collection, analysis, interpretation of the data, writing of the manuscript, or the decision to publish the results. Trial sponsors are the Bern University of Applied Sciences, Switzerland and the Vrije Universiteit Brussel, Belgium.

## DISCUSSION

This RCT will be the first sufficiently powered trial to investigate the efficacy and cost-effectiveness of adding a BWRP to best-evidence physical therapy (PNE plus CTET) in people with CNLBP and comorbid overweight or obesity in two different countries. Given the strong link between CLBP and overweight/obesity, as well as their detrimental bidirectional impact, this study will have the potential to significantly improve clinical care for people with CNLBP.

Despite the rise of medications facilitating weight loss, the cornerstone of obesity management in the United States, United Kingdom, and Europe lies in a multicomponent lifestyle intervention, including nutrition, exercise, and behavioral therapy.^77^^,^^78^ While anti-obesity medications are effective in supporting weight reduction, they are also associated with various side effects and adverse events that should not be underestimated.^77–82^ Moreover, access to these medications remains limited, not all patients are eligible to obtain them, and they are typically prescribed alongside mandatory lifestyle and dietary changes.^77^^,^^83^ Discontinuation of the pharmacotherapy results in an increase in body weight, indicating in a long-term dependency.^83^ Notably, combining physical activity with medication has been shown to improve healthy weight maintenance after treatment termination, compared to either intervention alone.^84^ Both Switzerland and Belgium follow a similar patient-centered, multidisciplinary approach as the first line of obesity treatment. In Switzerland, initial management includes dietary changes, increased physical activity, psychological support, and diabetes counseling when necessary.^85^^,^^86^ Only adults with a BMI ≥35 or a BMI ≥27 in combination with a comorbidity like high blood pressure may be eligible for anti-obesity medication.^85^^,^^86^ Similarly, in Belgium, policy recommendations emphasize treatments by a psychologist, dietician, and physical therapist, with anti-obesity medication considered as an optional component of treatment.^87^ This underscores the rationale for the planned RCT, which aims to evaluate the combined effects of PNE, CTET, and BWRP within a physical therapy setting in two countries.

The findings of this study can influence decision making by clinicians, policy makers, and other relevant stakeholders, and allow implementation of new health care pathways when treating such patients with CNLBP and overweight or obesity. If the new intervention corroborates the promising results of the proof-of-concept studies, it will have a high socioeconomic impact. Furthermore, the new combined treatment, incorporating a healthier lifestyle and higher physical activity to existent treatment, is expected to increase life expectancy in the long-term, as it is effective in reducing chronic health risks.^88^ Given the potential clinical and socioeconomic impact of the intervention, evaluating its cost-effectiveness is essential, as such economic assessments typically serve as the basis for implementing new treatment options in clinical practice. By focusing not only on clinical costs but also on cost-effectiveness, the intervention’s impact on health care consumption and work productivity can be assessed. While the former is relevant to health insurance companies, the latter holds significance for society and the economy. Current evidence indicates that productivity loss is a significant cost contributor for individuals with CLBP.^89^^,^^90^ By incorporating this evaluation into the present study, a comprehensive assessment of the intervention’s effects can be conducted, extending beyond clinical outcomes.

Currently, lifestyle factors such as a healthy diet, weight management, smoking cessation, and stress management are not consistently integrated into physical therapy curricula, and lack standardized education delivery across different countries.^91^ Therefore, establishing competence levels and standardized training within primary professional education is warranted. This is particularly important for integrating weight management, in addition to pain management strategies, for adults with CNLBP and overweight or obesity through physical therapists in the future. Given the importance of promoting physical activity and the need for a holistic approach to pain management, physical therapists are ideally positioned to implement these strategies as part of their clinical role.^92^ Until these competencies are fully incorporated into physical therapy curricula, additional educational programs could be used to equip practitioners with the necessary skills in the meantime. An RCT from 2023 demonstrated a significant improvement in physical therapists’ confidence, knowledge, and skills in weight management following an online education program on biopsychosocial elements of obesity and weight management.^93^ This provides evidence that such educational interventions are effective in enhancing physical therapists’ competencies in this area. Furthermore, a perspective paper from 2022 outlines how physical therapists can expand their professional scope and clinical decision-making, improve entry-level education, and stimulate new research in nutrition care and pain management.^94^ To ensure methodological transparency and facilitate accurate replication and future implementation in clinical practice, detailed intervention protocols from this RCT are provided in the Supplementary material.

This RCT has several strengths. The different recruitment strategies will allow to recruit a diverse and relevant study population. Moreover, a broad set of outcomes is used, enabling relevant multimodal assessment of participants. The trial is triple blinded, with assessors, patients and statisticians blinded for group allocation. Additionally, efforts will be made to guarantee the highest quality of the interventions, including extensive therapist training of a personalized approach within a standardized framework, availability of detailed treatment manuals, fidelity checks of audio recorded therapy sessions and assessment of therapy adherence.

There are some limitations to this study. A relatively high drop-out rate of 25% is expected due to the combination of education with exercise therapy, plus a weight reduction program. However, this is accounted for the sample size calculation, and efforts will be made to reduce drop-out. Additionally, the BWRP will be delivered by physical therapists without established experience in delivering this intervention. Yet, available training protocols, treatment manuals, and feasibility checks of therapy sessions should ensure highest treatment quality. Although some sociodemographic factors will be collected, data on ethnicity will not be included, which limits the ability to explore ethnic disparities across intervention groups and may impact the ability to fully assess the success of the randomization process. Nonetheless, the randomization procedure is assumed to deliver balanced intervention groups for any (un)measured variable.

### Data Access, Ancillary and Post-Trial Care

The research data will be archived in restricted access. Open Access will not be possible due to the high degree of confidentiality of the coded medical data, although all reasonable requests regarding sharing the research data will be considered by the researchers involved. The scientific project coordinators (first A.M., later W.V.B.), closely supervised by the principal investigators (J.N. and J.T.), will be responsible for data management. After the project, the data will be archived in the VUB (Vrije Universiteit Brussel, Brussels, Belgium) University Archive. The university server is equipped with user-level access permission management. The personal data of participants will be coded, and system encryption will be used to protect the coded data. To ensure data protection, all self-reported questionnaires will be collected and managed using REDCap.^95^ We will adhere to the principle of preservation of data and the policy of the VUB, which is to use a preservation term of 10 years. This is in accordance with the GCP-guidelines. The data in Switzerland will only be used in a coded version.

### Dissemination Policy: Trial Results, Authorship, Reproducible Research

Results of the study will be disseminated via national and international conferences and scientific journals. According to the International Committee of Medical Journal Editors (ICMJE) author guidelines, all publications will be planned and accomplished according to the individual contribution of the research team. Each single contribution will be planned with a publication agreement that has to be signed by all researchers involved.

## CREDIT—CONTRIBUTOR ROLES

Melanie Liechti (Conceptualization [equal], Methodology [equal], Project administration [equal], Visualization [equal], Writing—original draft [equal], Writing—review & editing [equal]), Alexander Philipp Schurz (Conceptualization [equal], Methodology [equal], Project administration [equal], Writing—review & editing [equal]), Arturo Quiroz Marnef (Conceptualization [equal], Methodology [equal], Project administration [equal], Writing—review & editing [equal]), Jan Taeymans (Conceptualization [equal], Funding acquisition [equal], Methodology [equal], Supervision [equal], Writing—review & editing [equal]), Ron Clijsen (Conceptualization [equal], Funding acquisition [equal], Methodology [equal], Supervision [equal], Writing—review & editing [equal]), Heiner Baur (Conceptualization [equal], Funding acquisition [equal], Methodology [equal], Supervision [equal], Writing—review & editing [equal]), Nathanael Lutz (Conceptualization [equal], Funding acquisition [equal], Methodology [equal], Supervision [equal], Writing—review & editing [equal]), Tom Deliens (Conceptualization [equal], Funding acquisition [equal], Methodology [equal], Supervision [equal], Writing—review & editing [equal]), Peter Clarys (Conceptualization [equal], Funding acquisition [equal], Methodology [equal], Supervision [equal], Writing—review & editing [equal]), Jo Nijs (Conceptualization [equal], Funding acquisition [equal], Methodology [equal], Supervision [equal], Writing—review & editing [equal]), Matteo Vanroose (Conceptualization [equal], Methodology [equal], Project administration [equal], Writing—review & editing [equal]), Wouter Van Bogaert (Conceptualization [equal], Methodology [equal], Project administration [equal], Supervision [equal], Writing—review & editing [equal]), and Anneleen Malfliet (Conceptualization [equal], Funding acquisition [equal], Methodology [equal], Project administration [equal], Supervision [equal], Writing—original draft [equal], Writing—review & editing [equal])

## CLINICAL TRIAL REGISTRATION

This study was registered on ClinicalTrials.gov (NCT05811624) on March 14, 2023.

## Supplementary Material

2024-0814_R1_FINAL_Supplementary_file_Study_Protocol_BO2WL_v2_pzaf097
