# Supplementary material for: Lifestyle Intervention in People With Overweight and Obesity and Chronic Low Back Pain: Study Protocol for an International Multicenter Randomized Controlled Trial
Source: Phys Ther. 2025 Aug 13;105(9):pzaf097. doi: 10.1093/ptj/pzaf097 (PMC12448435; doi:10.1093/ptj/pzaf097)
Supplement: 2024-0814_R1_FINAL_Supplementary_file_Study_Protocol_BO2WL_v2_pzaf097 [file 2024-0814_r1_final_supplementary_file_study_protocol_bo2wl_v2_pzaf097.pdf]

Lifestyle Intervention in People with Overweight and Obesity Suffering from Chronic Low Back Pain: Study Protocol for an International Multi-center Randomized Control Trial

Content

**Supplementary file 1: Recruitment strategies for study participants**	2
**Supplementary file 2: Details on the content and organization of the control intervention**	4
**Supplementary file 3: Details on the content and organization of the experimental intervention**	13
**Supplementary file 4: Training of therapists**	18
**Supplementary file 5: Therapy adherence and adverse events**	20
**Supplementary file 6: Fidelity criteria**	22
**Supplementary file 7: Clinical Assessments**	25
**Supplementary file 8: Food diary**	29
**References**	36

Supplementary file 1: Recruitment strategies for study participants

In both countries flyers and/or posters were created, which contain information about the study procedure, measurements, contact information of the study team and a link and/or QR-code to the project specific website (BE: https://www.vub.be/nl/nieuws/respondenten-gezocht-obesitas-en-chronische-lage-rugpijn and CH: https://www.bfh.ch/gesundheit/de/themen/personen-uebergewicht-rueckenschmerzen-gesucht/). No details on the therapy content are provided on the flyers or the project website. For Switzerland, posters are placed in the Bern University of Applied Sciences and medical institutions such as general practitioner's practices. Further, flyers are sent to all general practitioners and other health care providers in the city of Bern. For Belgium, patients are recruited from the University Hospital Brussels by distributing flyers. Further, all family physicians working within a 20-minute travel distance from the participating hospitals were contacted personally. Upon request, additional information about the project is given by telephone or in person. In addition, advertisements and announcements in local newspapers, pharmacies and online or printed publications from patient support groups and health insurance funds will be used as additional recruitment strategy.

Moreover, a social media campaign was launched in both countries using attracting pictures or a short video with captions to make the project visible on Facebook® and Instagram®. This campaign targets people from the study's predefined age range and interested persons who click on the link in the video, or the pictures are forwarded to the project website.

Lastly, the researchers work closely together with doctors and other health care professionals within the involved therapy centers. To those getting in contact with potentially eligible participants, the study is explained in detail, so they can distribute flyers among possible eligible patients.

Swiss physical therapists were recruited via an article in the newsletter of Physiobern, the cantonal association representing the interests of physiotherapists in the canton of Bern and the university hospital of Bern (Inselspital) has been contacted to recruit interested physical therapists. In Belgium therapists were recruited via a vacancy that was launched and distributed via the Flemish Universities and alumni social media channels.

Supplementary file 2: Details on the content and organization of the control intervention

Detailed information about the procedure and content of the control intervention are displayed in Table 1 to 3.

Table 1 Overview of the practical organization of the control intervention, including the frequency and specific content of the sessions per week.

**Weeks**	**Content of the session**	
Week 1	Pain Neuroscience Education (one-on-one session)	Pain Neuroscience Education (online session)
Week 2	Pain Neuroscience Education (one-on-one session)	
Week 3	Cognition-targeted Exercise Therapy	Cognition-targeted Exercise Therapy
Week 4	Cognition-targeted Exercise Therapy	Cognition-targeted Exercise Therapy
Week 5	Cognition-targeted Exercise Therapy	Cognition-targeted Exercise Therapy
Week 6	Cognition-targeted Exercise Therapy	
Week 7	Cognition-targeted Exercise Therapy	
Week 8	Cognition-targeted Exercise Therapy	
Week 9	Cognition-targeted Exercise Therapy	
Week 10	Cognition-targeted Exercise Therapy	
Week 11	Cognition-targeted Exercise Therapy	
Week 12	Cognition-targeted Exercise Therapy	
Week 13	Cognition-targeted Exercise Therapy	
Week 14	Cognition-targeted Exercise Therapy	

Part 1 – Pain Neuroscience Education (PNE)

Each participant in the control group will receive 3 sessions of Pain Neuroscience Education, of which two (one-on-one and one online) sessions are planned in the first week and a third session is carried out in the second week. The detailed content of these sessions is outlined in detail in Table 2. Therapists were equipped with manuals and all necessary tools, such as a PowerPoint presentation and informational leaflets, to effectively deliver pain neuroscience education (PNE) and adhere closely to the protocol.

The aim of PNE is to reconceptualize pain by converting knowledge on the development and processing of pain, and to increase pain coping skills. Participants

will learn that all pain is eventually a product of the brain, and that hypersensitivity of the central nervous system, rather than local tissue damage contributes to their symptoms. PNE enables patients to understand the controversy surrounding their pain, including the lack of objective biomarkers or imaging findings. The content, format and pictures in the sessions are based on available handbooks,[1,2] which are already successfully used in previous studies for treatment in patients with CLBP [3,4].

Part 2 – Cognition-targeted Exercise Therapy (CTET)

Once adaptive beliefs regarding CLBP are acquired through PNE, a time-contingent, cognition-targeted approach to daily activity and exercise therapy will be applied. Important is the time-contingent approach, the continuous targeting of cognitions and perceptions about their problems and the outcome of each exercise and the gradual progression to more feared movements and activities. The specific content of the exercises is tailored based on the "feared activity form". "Cognition-targeted" implies the aim to change inappropriate beliefs and perceptions into correct ones while performing these feared activities. The purpose of this program is to confront the patient with movements and activities that are feared, avoided and/or painful. It is crucial to avoid all 'safety behavior' and to focus on normal and functional movements. First exercises only induce a limited amount of fear or stress, and progression is targeted and developed towards those movements and activities the patient avoids or is fearful of (e.g., bending forward). The program will also include a home exercise program, which will be composed by exercises the participant felt confident performing at home. Patients' cognitions and perceptions about their problem and about exercises will be addressed throughout the treatment. Further details can be found in published work [5] and Table 2. Therapists were equipped with manuals and all necessary tools,

such as communication sheets, a step-by-step overview of the CTET approach (Table 3), to effectively deliver CTET and adhere closely to the protocol.

Table 2 Description and structure of PNE and CTET in the control treatment arm

**Pain Neuroscience Education**		
Session 1	On site, 30 min	The first session is an **individual session**, where the patient and therapist go over an interactive presentation together. This presentation includes the following topics: the characteristics of acute vs chronic pain, how pain becomes chronic (including plasticity of the nervous system, pain modulation, central sensitization), and potential sustaining factors such as emotions, stress, cognitions and behavior.<br>At the end, the patients receive a leaflet containing the same information as the presentation, with the instruction to re-read the information in the upcoming days and have it read by a significant other if possible.<br>Participants are also asked to fill out the “feared activity form” and to bring it to the next session (for an example, see [5]). The feared activity form is based on individual activities of which the patient believes they negatively impact and/or aggravates pain symptoms. The patients have to list these activities and rate how convinced they are, that the activity or movement will worsen their pain on a scale from 0 to 10. This form will be used to develop an individually targeted exercise therapy (part 2, below) for activities or movements that matters to the patient.
Session 2	Online, at home	The second session is an **online module** (available at https://sites.google.com/view/onlinemoduleweave/home) performed at home. The patient receives a link via mail and can browse through the slides at their own pace.<br>On the same webpage the patient is requested to fill out a questionnaire, which monitors their understanding of pain and the content of PNE and questions their belief that the content is applicable to their situation. Their answers serve as guideline for the third individual session with the therapist.
Session 3	On site, 30 min	The 3rd session is an **individual session** addressing the patient’s questions and translating the content of the first session and the online module to daily life. According to the questionnaire, which was completed during the online session by the patient, an individual reflection and discussion on pain beliefs can be carried out. In general, perceptions are questioned, goals are discussed, and therapeutic alliance is checked. For this reason, a vicious circle layout (focusing on the thoughts, emotions and behavior linked to pain) will be used and individually filled with the patient, to enhance comprehensibility of the therapy concept in their specific case.<br>Goals must be functional goals (not focused on pain reduction) and they are written down in according to the SMART principle: specific, measurable, achievable, realistic, timely.
**Cognition-Targeted Exercise Therapy (CTET)**		
Session 4-17	On site, 30 min	During the first 14 CTET sessions, the following information applies:<br>With the usage of the feared activities form and the predefined goals exercises are selected to build up the feared movements from less to more feared movements or activities. An example exercise catalog is provided to the therapists, but they are encouraged to also use their own exercises and adapt it to patient’s preferences. The therapist gradually increases towards more feared activities over time and adds new exercises to achieve the feared activities.<br>A “step-by-step approach” (Table 3) including communication for each situation is available for the therapist, as the communication for instruction and execution of the exercises is key. The approach pursues a time-dependent and not pain-dependent execution of the exercises. In a first step, the therapist explains what the exercise entails and then questions cognitions and perceptions of the patient. In the second step, the therapist demonstrates the exercise first, followed by the patient. After the exercise, consequences of the exercises are questioned and discussed if necessary. If the patient indicates pain during the exercises, the therapist checks whether the patient understands

		what the pain means and checks the understanding of the PNE sessions. If the patient still links pain to threat or damage, the therapists ask strategic questions to challenge the patient to reflect on their pain beliefs, and to implement the knowledge gained during the pain education sessions. If needed, specific content of this education can be repeated briefly.<br><br>Home exercises are given only if the patient was able to perform them successfully during the therapy and includes a good communication about the approach of the exercise at home and how the patients must react if the pain flares up.
Session 18	On site, 30 min	In the last session, the therapist reviews the initial goals that were written down during the last education session and discusses the patient's progress toward achieving them. They identify what has gone well, where challenges have arisen, and what the patient aims to accomplish in the follow-up period and long term.<br><br>Patient and therapist select exercises for continuation after treatment finalization together and the therapist provides detailed instructions to ensure the patient can perform them independently. If possible and/or required, additional exercises for the coming weeks or months are discussed and planned, focusing on which exercises will be performed, how often, and strategies for overcoming potential obstacles.

Table 3 Step-by-step overview of the CTET approach

Prerequisite to start

Before starting exercise therapy, there should be therapeutic alliance around both pain education and the behavioral strategy for the exercise program.

Actions and prior communication.

The Feared Activities Form lists the activities the patient is afraid of. Goals are formulated with the patient based on these listed activities. The Feared Activities Form is given at the first pain education session (see checklist) and returned completed by the patient during the first practice session (session 4). If there was enough time in the third education session, the goals were already established, and exercise therapy can be started. If this has not yet happened, the goals are determined at the start of the first exercise therapy session.

Communication to patient before starting exercises

- I would like to do exercises with you despite the symptoms you have. I want to make you experience that you can do all these activities and movements, despite your fear and pain. Are you comfortable with that? Of course, we will do that step by step. So, when there is pain, we will NOT stop the activity because we know that pain is not a reliable symptom anymore. We want to teach the brain again that exercise and movement is not bad. Are you comfortable with that?
- We are going to work time-contingent instead of pain-contingent. This means that we will no longer pay attention to the pain but will determine in advance how many repetitions or how long we will perform an exercise. Do you see that as possible? Why do you think we do it this way?
- Pain has no meaning to you. Your alarm system goes off without an intruder. Your medical condition does not prevent you from performing all possible activities, it is your alarm system that prevents it.
- Do you understand how this program is going to affect the hypersensitivity of your nervous system?

General guideline:

Cognition-targeted exercise therapy - general principles and guideline.

1. **All exercises are performed TIME-contingent (NOT pain-contingent)**
2. Explain what the exercise entails.
3. Question cognitions and perceptions and discuss if necessary.
4. Therapist demonstrates exercise.
5. Patient performs exercise.
6. Question consequences of exercise and discuss if necessary.

➔ Important not to focus on pain!

If the patient indicates pain when performing an exercise, it is checked whether the patient understands what this pain means. If the patient cannot give a clear answer to this or shows maladaptive cognitions and beliefs, the pain education is repeated again.

General principles of exercises

1. You can use any exercise, as long as it fits to and is applied within the cognition-targeted exercise principles, it is linked to the goals and feared activities of the patient (and this link is also made clear to the patient) and it fits within a gradual structure. For inspiration we also have a document with all kinds of example exercises.

2. When one of the problems is 'prolonged sitting or standing', it is especially important to bring movement into the static postures (NOT the best or most ergonomic posture, these things are outdated). So, it comes down to getting your patient to move the back regularly, in general but especially very specifically (e.g. by pelvic tilt - taught in sitting and standing, does not have to be a 'perfect' execution).
3. Progression within 1 exercise can be made via:
    a. Different starting position: lying - sitting - standing
    b. Stable vs. unstable surface (Airex matress, sitting ball)
    c. Number of repetitions
    d. Duration of static exercises
    e. Adding or increasing weight
    f. Single (e.g. flexion only) to more complex movements (e.g. combination rotation and flexion/extension)
4. Home exercises are given only if the patient was able to perform them successfully in the session with the therapist, and if communication (see below) about them was good. This includes discussing with the patient how he/she will approach this and what he/she will do when a pain shooting occurs.

Communication manual for specific situations

T = Therapist / P = patient

1. **At the beginning of treatment**

    T: I understand from our previous session and the activity form you filled out that you no longer perform [certain movement/activity]. Why is that?

    P: Gives a biomedical reason.

    T: The reason you give was from before you received information about the changes and hypersensitivity of your body and nervous system. Now you know that pain and damage do not equal each other and that the information from the muscles and joints coming into your brain have been amplified. How do you currently stand in relation to that new information?

    P: I understand that, but the wear and tear in my back is still there, isn't it?

    T: Many persons of your age have such wear and tear in the back, but few suffer or have pain with it. So, what does that say about the wear and tear in your back?

    P: That it is not so bad?

    T: Indeed, it does. To further reassure you: before you participated in this study, you were extensively examined and questioned. From that information, I know that you shouldn't worry about that at all. The exercise you will do here and perform at home are perfectly safe for you.

2. **Pelvic tilt (sitting - hands / knees - stance)**
    T: Through your activity form, together we have established a number of goals that we want to work towards with this treatment. In order to achieve these goals, we have to work systematically and start with a number of exercises of which you will not always immediately see the link with the predetermined activities. Do you understand that?
    P: Yes.
    T: Well, the first exercise we can start with will be such an exercise. It involves tilting the pelvis forward and backward. Do you think you will be able to perform this exercise?
    P: I don't know, I don't think that will go smoothly right away.
    T: Of course. That's also not abnormal when you haven't just moved for a long time. Shall we give it a try together? In stance/al sitting/...? I will demonstrate the exercise and then you can try.
    P: Ok
    T: (demonstrates exercise for) What do you think will happen when you perform this movement?
    P: That the pain will increase.
    T: And what does that mean when the pain increases? Does that have significance for your muscles, for your joints,...?
    P: No, I know I shouldn't pay attention to the pain I will get.

T: Good. Then you may performe this movement.
T: How did the exercise go?
P: Good, less frightening than expected.
T: Very good. So, turns out your expectations were worse than the actual consequences. That's very good, that can give you confidence for the next exercises!

When the pain is indeed increasing, P can say, "You see the pain is increasing."
T: It's about what meaning you give to this pain. What do you think it means now?
P: That something has gone wrong in my neck/back?
T: Again, that's an idea from before all the pain education you received. When you look back at all the previous information around pain that you've received from me, what can you say about that pain now?
P: That it's my nervous system. That my alarm goes off without an intruder inside?
T: Right. So, what are we especially not allowed to do?
P: Listening to the pain. I have to keep moving despite the pain.

3. **Functional exercises**

T: The past exercises were preparatory to the activities we set as our goal for this treatment. Today we will also start adding some more functional exercises. Let's look at the goals we made up together. I'm looking here e.g. at [goal 1]. If we break this down, we can say that this activity consists of several elements, such as [functional exercise 1], [functional exercise 2] and [functional exercise 3]. I would like to practice each of these elements separately with you, to eventually merge this into the overall activity. Can you follow me in that?
P: Yes
T: Which of these elements causes you the least concern?
P: Indicates the exercise/movement
T: Then shall we start with that? Do you think you are able to perform that exercise partially or fully?
P: I don't know, I don't think that will go smoothly right away.
T: Of course. That's also not abnormal when you haven't just moved for a long time. Shall we give it a try together? In stance/al sitting/...? I will demonstrate the exercise and then you can try.
P: Ok
T: (demonstrates exercise) What do you think will happen when you perform this movement?

➔ Continue see point 2

4. **Home exercises**

T: Let's talk a little bit about home exercises. You can perform these exercises perfectly here in practice. Are you willing to perform these exercises at home as well? Without help and guidance from me?

P: Yes

T: If we calculate that one training session consists of 3 sets of 15 repetitions, how many times a week are you willing to practice?

P: Every day? Every other day?

T: How about exercising 6 times a week, giving you 1 day off? Is that good for you?

P: Ok.

T: It's unlikely, since you're doing so well here, but suppose the exercises at home don't go so well. Suppose you're halfway through exercising and suddenly you feel a shooting pain in your back. What will you do then?

P: Stop and try again later?

T: Does that shooting pain mean there is something wrong with your muscles and joints?

P: No, my body's alarm goes off without anything being wrong. The shooting pain is there only because my body is hypersensitive.

T: Very correct. The sensitive alarm system should not stop you from doing exercises and should not stop you from taking control of your own life again. You can compare it to rewarding a child when he or she

exhibits bad behavior: canceling the exercise because of the pain causes you to reward your brain for producing pain and we don't want that.

5. Exercise stopped because of pain

T: I see from your exercise journal that you stopped your exercise. Why is that?

P: I stopped because it hurt too much.

T: What do you think would happen if you continued the activity anyway when in pain?

P: That I would injure my back.

T: Linking back to the lessons around pain, what did you remember from that?

P: That pain and damage do not equal each other.

T: Correct, so you know that this is true. What information did you get from the past tests and imaging you underwent before starting this treatment?

P: That there is indeed nothing wrong with my muscles and joints.

T: Then how come you stopped exercising anyway? Don't you have confidence in the story I told you?

P: Yes, it's just difficult to put this into practice.

T: Good, then we are on the same page. We both agree that there is no harm, and your body/brain is reacting hypersensitive. I suggest that we redo the exercises you were going to do at home here, so that you regain confidence that you can do this at home.

6. Increase in pain

T: You indicate that you get pain with this exercise. What do you yourself think is the reason for this?

P: Indicates biomedical reason.

T: The reason you give was from before you got information about the changes and hypersensitivity of your body and nervous system. Now you know that the information you get from your muscles and joints is amplified. How do you feel about that information right now?

P: I understand it, but I still feel a little uncertain about it.

T: That makes sense. The best way to gain confidence in this information is to do the exercises anyway despite the pain. In this way, you will find that you can actually do the exercises, and you will gain more and more confidence in your body and abilities.

T: If you ignore the pain for a long time, what do you think would happen?

P: I think I would feel this less over time.

T: You say "in time," so you are indicating that this will not be the case immediately?

P: Indeed, I have been in pain for so long, I don't think this will diminish any time soon.

T: I can follow you in that. Then can you also follow me when I say that we should ignore the pain and move despite the pain? So that the pain becomes less significant in your life?

P: Yes.

7. Sudden flare-up after long time progression

T: I understand that this must be very frustrating.

P: Indeed, it is.

T: What cause do you give to this yourself? What do you think is the reason for this sudden flare-up?

P: That I have been practicing too much.

T: Then what exactly do you mean? Then what effect did those exercises have on your body?

P: I think I damaged my back.

T: Why do you think that? Why could exercises that are perfectly safe still cause damage?

P: I don't know.

T: Do you see it as possible that it was your alarm system that suddenly went back into action for a while?

P: I don't know.

T: As you know, we never set as a goal that the pain would diminish. So, a flare-up is perfectly possible, and it is possible that they will still occur. However, it's all about what meaning you give it and how you deal with it.

Communication pitfalls.

1. You should not have the patient focus on specific conditions for moving to next step in exercise therapy.
2. Never set pain reduction as a goal, but rather focus on improving function and quality of life.
3. Do not get into a discussion with the patient: Do not impose own vision, patient must come to new insights himself.
4. Do not show fear when giving exercises. Do not show fear yourself when the patient complains of an increase in pain.
5. Do not focus on strength or endurance when communicating with the patient. You might have it as an underlying goal, but the patient should only focus on functional goals. When focusing on biomedical constructs as strength or endurance, possibly the patient will use this as an escape route: "I can't do it, I don't have the strength for it."
6. Make sure the patient has plenty of success experiences. Start with activities the patient enjoys.
7. The exercise should be performed long enough (enough repetitions) in the session together with you so that there is sufficient decrease in anxiety. E.g., to the point that the patient says he or she can perform it at home.
8. Ensure sufficient variation: avoid generalization and relapse.
9. When the patient exhibits anxiety, it is important that the patient identifies the catastrophizing cognition or belief.
10. Do not allow for safety behaviors or ergonomic principles (i.e. NO best posture, NO ergonomic posture, NO activation of stabilizing muscles).
11. Allow the patient to formulate new cognitions and perceptions by himself. Do not bring knowledge yourself but let it come out of patient via questioning.

Supplementary file 3: Details on the content and organization of the experimental intervention

Detailed information about the procedure and content of the experimental intervention can be found in Table 4 and 5.

Table 4 Overview of the practical organization of the experimental intervention, including the frequency and specific content of the sessions per week.

Weeks	Content of the session	
Week 1	Pain Neuroscience Education (one-on-one session)	Pain Neuroscience Education (online session)
Week 2	Pain Neuroscience Education (one-on-one session)	
Week 3	Behavioral weight reduction program, focused on nutritional habits	Cognition-targeted exercise therapy
Week 4	Behavioral weight reduction program, focused on physical activity and sedentary behavior	Cognition-targeted exercise therapy
Week 5	Behavioral weight reduction program, content depending on personal difficulties/aims and questions	Cognition-targeted exercise therapy
Week 6	Behavioral weight reduction program, content depending on personal difficulties/aims and questions	
Week 7	Cognition-targeted exercise therapy	
Week 8	Behavioral weight reduction program, content depending on personal difficulties/aims and questions	
Week 9	Cognition-targeted exercise therapy	
Week 10	Behavioral weight reduction program, content depending on personal difficulties/aims and questions	
Week 11	Cognition-targeted exercise therapy	
Week 12	Behavioral weight reduction program, content depending on personal difficulties/aims and questions	
Week 13	Cognition-targeted exercise therapy	
Week 14	Cognition-targeted exercise therapy and Behavioral weight reduction program: Long term planning.	

In the experimental treatment arm, the first two weeks, which include pain neuroscience education, are identical to the control intervention in both practical implementation and content. The cognition-targeted exercise therapy (CTET) component is also content-wise identical to the control intervention. However, as shown in Table 4, some CTET sessions were replaced with BWRP sessions. Additionally, as part of the behavioral weight reduction goals, this group focused on

increasing physical activity, which was also delivered using a cognition-targeted approach. Table 5 shows the content and structure of the specific Behavioral Weight Reduction Program (BWRP) sessions. Experimental therapists were equipped with manuals and all necessary tools, such as informational leaflets, infographics, communication sheets and FAQ's, to effectively deliver BWRP and adhere closely to the protocol.

Table 5 Description and structure of the experimental intervention

**Behavioral Weight Reduction Program**		
General		Using the principles of motivational interviewing, shared decision making and promoting self-management throughout the 15 sessions, patient and therapist will come to an individually tailored weight reduction program. The use of motivational interviewing aims to develop autonomous motivation by increasing perceived competence and self-regulation [6]. During every session the patients' actions with regard to the weight loss program will be evaluated, discussed and reinforced or tailored in more detail. Behavioral change principles of goal setting, self-monitoring, and feedback on the behavior [7] will be integrated throughout. Consequences of unhealthy lifestyle factors will be discussed, together with general encouragement and examples of how improving lifestyle factors can influence pain outcomes and quality of life [6].
Sessions 1 - 3	On site and at home	Pain Neuroscience Education, Identical to control therapy, see Table 2 for details.
Session 4	On site, 30 min	This is the first session of the BWRP and contains a general explanation of the therapy, its philosophy, what a healthy eating pattern is, and the food triangle is introduced. In a second step, patients' food habits are screened using the food diary (which is filled out as part of the baseline assessment). According to the food habits three challenges are introduced to the patient:<br>1. Challenge screening eating habits part I<br>(Screening eating behavior according to the food diary)<br>2. Challenge fruits and vegetables<br>(Aim to increase the fruit and vegetable intake)<br>3. Challenge goal setting<br>(Defining goals regarding nutrition/habits)<br>A specific treatment manual is available for the content of the first session, including infographics of the food triangle and a priori developed documents for the challenges.<br>If possible, challenge 3 can be implied in the therapy or if there is not enough time, the patient can take it home, fill it out and bring it back to the next therapy session for discussion.
Session 5	On site, 30 min	Cognition targeted exercise therapy, identical to control therapy, see Table 2 and 3 for details.
Session 6	On site, 30 min	This is the second session of the BWRP and contains the role of physical activity and sedentary behavior. Again, a detailed treatment manual is available for the therapist.<br><br>The session contains:

		1. Evaluation of integrating the new behavior of last week<br>2. Small recap of the nutritional education<br>3. Why physical activity is important<br>4. Difference between physical activity and exercising<br>5. Guidelines physical activity & sedentary behavior: the moving triangle and rate of perceived exertion scale (RPE).<br>6. Challenge Goal Setting Physical Activity<br>(Defining goals regarding physical activity/ sedentary behavior)<br>7. Challenge action & coping planning<br>(Planning of becoming more active and how to overcome potential barriers)<br>8. Fitbit<br>(Explanation of handy functions of the Fitbit watch, to increase motivation for physical activity)<br><br>First, the therapist asks open questions to the patient, how their general impression of the therapy is, how the last week has been and if they have faced any barriers or problems. In addition, the challenges 1-3 are discussed and any questions of the patients are addressed. In a second step, a short recap of the topics of the previous sessions is given.<br><br>Point 3-5: The therapists explain the importance of physical activity, the difference between physical activity and exercise and the guidelines for physical activity & sedentary behavior. For a better understanding, a factsheet of the moving triangle and the rate of perceived exertion scale are shown, and examples are collected with the patient. Any questions are discussed and then the next challenges are introduced.<br><br>Point 6 &7: In a shared decision-making process, the patient defines at least one specific goal for physical activity & sedentary behavior. The therapist explains some potential barriers and solutions to overcome barriers. In this way the challenge of action & coping planning is introduced to the patient. This challenge prepares the patient for potential difficulties which may emerge during the implementation of their defined physical activity and how to overcome it. Further, a weekly plan to structure the activities might help to schedule the activities on convenient days or timepoints. Specific documents are available for the challenges.<br><br>As a last step, some functions of their Fitbit watch are explained to motivate the patient. Therefore, the function as an activity tracker is explained and how personalized notifications for daily steps or activity minutes can be programmed.
Session 7	On site, 30 min	Cognition targeted exercise therapy, identical to control therapy, see Table 2 and 3 for details.
Session 8	On site, 30 min	This is the third session of the BWRP and a re-assessment of the eating habits is performed.<br><br>The session contains:<br><br>1. Evaluation of integrating the new behavior of last weeks<br>2. Small nutrition recap, physical activity and sedentary behavior<br>3. Challenge screening my eating habits part 2<br>(Screening eating behavior according to the food diary)<br>4. Challenge whole grains<br>(Aim to replace non-whole grain products by whole grain products)<br><br>The session starts again with the evaluation of the integration of the new behavior of the last weeks and a brief repetition of the nutrition, physical activity and sedentary behavior is conducted. The patient is asked what

		they remember of the last session and what they think is the most important.<br>Then, the eating habits are screened again and the challenge “whole grains” is implied which contains the replacement of a non-whole grain product (e.g. white rice) with a whole grain product (e.g. brown rice) for at least one meal every day the next week. On the document of the whole grain challenge, the patient is advised to note down, where possibilities are seen to replace non-wheat grains (breakfast, lunch, dinner from Monday to Sunday).
Session 9	On site, 30 min	Cognition targeted exercise therapy, identical to control therapy, see Table 2 and 3 for details.
Session 10, 12, 14, 16	On site, 30 min	The fourth until the seventh BWRP sessions always include the same set-up:<br><br>1. Evaluation of integrating the new behavior of last weeks<br>2. Aspects and guidelines to consider for the rest of the therapy<br>3. Different challenges (see below)<br><br>Each session begins with an evaluation of how well the patient has integrated the new behavior over the past few weeks, how the adherence to the program is perceived, how barriers can be coped with and how the challenges were implemented. Celebrate successes throughout the therapy and focus on the positive aspects even if the patient did less than initially planned.<br>In addition, questions can be discussed and the patient is asked, for which part of the therapy he/she needs more guidance at the moment.<br><br>If possible the therapist can proceed to further challenges in agreement with the patient’s needs:<br><br>1. Challenge screening eating patterns Part 3<br>(Screening eating behavior according to the food diary)<br>2. Challenge legumes, nuts and seeds<br>(Aim to integrate some legumes, nuts and seeds in the diet)<br>3. Challenge fatty fish and fats<br>(Aim to integrate fatty fish once a week and to replace saturated fats by unsaturated variants)<br>4. Challenge eat less meat<br>(Aim to integrate one vegetarian day into the week)<br><br>Next to challenges, there exist also factsheets and tips of different topics:<br>1. Tips for doing groceries<br>(E.g. doing a grocery list, check food stocks)<br>2. Tips on how to become more active<br>(Tips to integrate physical activity in daily life)<br>3. Tips to lose weight<br>(E.g. tips for nutrition, sleep, physical activity)<br>4. Factsheet Nutriscore<br>(Explanation of the Nutriscore and how to use it)<br>5. Factsheet sports nutrition<br>(Triangle for sports nutrition)<br>6. Factsheet water<br>(Recommendation for daily water intake)<br><br>For all the challenges, the therapist has extra information on how to explain the challenge and to give some tips and examples for the implementation.

Sessions 11, 13, 15, 17	On site, 30 min	Cognition targeted exercise therapy, identical to control therapy, see Table 2 and 3 for details.
Session 18	On site, 30 min	This is the last session of the BWRP and the therapist reflects on the initial goals together with the patient.<br><br>There is a dialogue between the patient and therapist about the exercises, challenges and habits, which the patient feels comfortable continuing and the therapist provides a detailed description on how the patient can continue by his/her own.<br>Leading questions for this step:<br><br>1. What do you want to change?<br>2. How are you going to change?<br>3. When are you doing this? (Daily/weekly planning, but also long-term planning)<br>4. What/who can help?<br>5. Which barriers or problems are expected and how can they be handled?<br><br>The therapist may also send the challenges or extra information on diet or physical activity via e-mail directly after the last session.

Supplementary file 4: Training of therapists

To ensure simultaneous and equal training of all therapists in both countries, training is delivered online and in English. Training sessions are recorded and available online (in restricted access), so the therapists involved can rewatch the training sessions at any time. All therapists receive the same training related to PNE and CTET, while training on BWRP is only delivered to the experimental therapists. Table 6 shows the time-investment of the training and the content per training session. The total time-investment for control therapists was maximally 7h, while the additional time-investment for experimental therapists was about 8h.

In general, therapists are advised to report the therapy content similar to what they would do in normal day-care (using their laptops and templates). Once therapy had ended for a patient, they receive a link, allowing them to fill out general reflections on the therapy, patient adherence and compliance and potential adverse events. Every year a refresher course will be organized.

Table 6 Schedule of training of therapists

**Training session**	**Time-investment**	**Content**
At home preparation before training session 1	Max 2h30	1. Read information leaflet participants receive during the first PNE session.<br>2. Watch the online module for participants.<br>3. Watch a demonstration of a PNE session using the guiding Powerpoint
Online training session 1	Max 1h30	1. General information on study organization<br>2. Background on PNE, including philosophy behind it and evidence.<br>3. Overview of available tools and manuals and use of study platform (SharePoint)<br>4. Instructions on audiotaping sessions for fidelity assessments<br>5. Q&A
At home preparation before training session 2	Max 1h30	1. Get familiarized with the study platform (SharePoint)

		2. Watch an instructive video on CTET, including a short recap on PNE, information on the fear-avoidance model, the evidence for CTET, the general principles of CTET, and available manuals and tools on the study platform (SharePoint)
Online training session 2	Max 1h30	1. Recap of PNE<br>2. Some communication examples.<br>3. Basic principles of motivational interviewing<br>4. Study documents related to CTET.<br>5. Case study of CTET<br>6. Q&A
Online training session 3 (limited to experimental therapists)	Max 2h	1. General principles of the BWRP related to nutrition, including governmental guidelines, EAT-Lancet guidelines, the food triangle, motivational interviewing, goal setting, etc.<br>2. Structure of SharePoint files (tools, manuals, challenges, factsheets) related to BWRP.<br>3. Q&A
At home preparation before training session 4 (limited to experimental therapists)	Max 4h	1. Study all BWRP files on the study platform (SharePoint) in detail.<br>2. Reflect on your current practice
Online training session 4 (limited to experimental therapists)	Max 2h	1. General principles of the BWRP related to physical activity and sedentary behavior, including (governmental) guidelines, goal setting, action and coping planning, use of Fitbit, etc.<br>2. Content of other BWRP sessions<br>3. Q&A

Supplementary file 5: Therapy adherence and adverse events

To record therapy adherence of the participants, physical therapists are asked to document the content of the therapy sessions as usual in their daily working routine. By the end of the 14-week therapy period, the therapists are asked to fill out a questionnaire on therapy adherence, compliance, and adverse events. The same questionnaire was used for control and intervention therapists, and includes the following questions:

1) Indicate the number of therapy sessions provided within the 14-week period (maximally 18, including the one online session)
2) Indicate how many sessions were spend at each part of the intervention (i.e., number of sessions PNE, number of sessions CTET, and number of sessions weight reduction if applicable)
3) What was the total duration (in weeks) of therapy?
4) What was the total duration in weeks spend at PNE sessions?
5) What was the total duration in weeks spend at CTET sessions?
6) What was the total duration in weeks spend at weight reduction sessions (if applicable)?
7) Did any of the following adverse events occur during therapy:
    a. Increased pain
    b. Cardiovascular issues
    c. Psychological issues
    d. Adherence challenges
    e. Nutritional deficiencies
    f. Weight cycling
    g. Other, being ….
    h. None

8) If adverse events occurred, please provide details related to it.
9) Please rate the participant's overall compliance of the whole therapy on a scale of 0-10 (0= absolutely no adherence; 10= perfect adherence). Optional: Add any comments related to adherence.
10) Only for experimental therapists: Please rate the participant's overall compliance of the weight reduction therapy on a scale of 0-10 (0= absolutely no adherence; 10= perfect adherence). Optional: Add any comments related to adherence.

Supplementary file 6: Fidelity criteria

The fidelity criteria are developed separately according to the treatment content of PNE, CTET and BWRP, containing the following items:

Part 1 – Pain Neuroscience Education

1) Explanation of pain (by patient)
2) Application of shared decision making
3) Balance of questions (open-ended, reflective, confrontive)
4) Attitude of therapist (respectful and nonjudgmental)
5) Recapitulation of previous session
6) Customization of PNE to patient's needs
7) Usage of change talk
8) Usage of layman's words and metaphors
9) Opportunities for patient to ask questions
10) Adherence to 30min timespan
11) Adherence to one-to-one format
12) Handout of leaflet
13) Explanation of acute and chronic pain
14) Difference between damage and pain
15) Discussion of peripheral and central sensitization
16) Discussion of neurophysiological aspects
17) Discussion of causes of chronic pain

Part 2 – Cognition-targeted Exercise Therapy and Behavioral Weight Reduction Program

1) Check of learning objective
2) Application of shared decision making
3) Balance of questions (open-ended, reflective, confrontive)
4) Attitude of therapist (respectful and nonjudgmental)
5) Recapitulation of previous session
6) Usage of change talk
7) Review of homework
8) Stimulation of integration of new behavior
9) Application of individually tailored program
10) Adherence to 30min timespan
11) Alternating CTET and BWRP sessions

Content CTET

12) CTET according to predefined goals
13) Exercise delivery time-contingent
14) Targeting cognitions during exercises
15) Avoiding safety behavior

Content BWRP

16) Physical activity and change in dietary behavior
17) Definition of goals
18) Explanation of “the holy three”
19) Start of new behavior
20) Explanation of food triangle

21) Challenge “screening eating patterns” instructed

22) Explanation of physical activity triangle

23) Importance of physical activity

Supplementary file 7: Clinical Assessments

A case report form was developed to ensure a standardized procedure for all assessors in both countries during all on-site assessment timepoints. Further, a template was developed to ensure appropriate data collection of the ultrasound and circumference measurement, including marking points, device settings and reference pictures. The ultrasound measurement points were identified according to the International Society for the Advancement of Kinanthropometry (ISAK) standards for anthropometric assessments [8]. More detailed information on each assessment can be found below.

1. Details on Body Composition Assessment – Tanita

Before body composition is assessed, the height of participants is measured using a SECA stadiometer (SECA, Hamburg, Deutschland). To assess body composition, the TANITA Bio-electrical Impedance Analyzer (TANITA MC-780SMA, TANITA Europe B.V., Amsterdam, Netherlands) is used. The Tanita gives a broad range of body composition outcomes, of which the following will be used in the current trial: BMI (kg/m^2), body fat (%), muscle mass (%), body water (%), and fat free mass (%). Bio-impedance analysis is a convenient, low-cost, non-invasive and reliable tool to assess body composition [9].

2. Details on Fat Distribution – Waist Hip Circumference

Waist and hip circumference are assessed using the Cescorf measuring tape. Each point is assessed twice, in between the assessor steps aways from the participant. If these two assessments vary more than 0.4mm, a third assessment is taken. In case

of two measurements, the mean is calculated for the analysis. In case of three assessments, the median is included in the analysis.

To assess waist circumference, the participant is standing on a stepper and crossing arms. The researcher is facing the front of the participant. Circumference is measured at the point of minimal circumference (between lowest ribs and crista). The participant is instructed to breathe normally, the measure is taken at the end of relaxed expiration. If no curvature is visible, the measurement is performed at the height of the belly button.

To assess hip circumference, the participant is also standing on a stepper while crossing arms. The researcher is facing the side of the participant. Hip circumference is measured at the maximal circumference (i.e. symphysis, biggest area of the gluteus muscle).

3. Details on Ultrasound assessment

Ultrasound (US) assessments are performed using the MicrUs EXT-1H REV:D ultrasound scanner with B-Mode/ M-Mode and Pulsed-Wave Doppler feature (TELEMED Ltd, Vilnius, Lithuania) with a linear probe (40mm scan with, 5-12MHz frequency range). Standard device settings are: Depth 50mm; Focus 14-18mm; Gain 40-66% but is adapted depending on the subject subcutaneous fat tissue thickness to gain best quality. When appropriate a gel spacer is used, except for the assessment at the multifidi site. The probe is placed perpendicular to the skin and assessors do not apply pressure on the skin. Prior to the US assessment, the right side of the body is marked based on different landmarks, according to the ISAK guidelines [8]. All measurements are performed on the right side only. For each test site maximally 2 frames are stored. Test sites include the following landmarks:

I. **Biceps:** For the measurement the participant is lying on the back, pillow under head and knee, probe is placed longitudinally, pointing towards the head.

II. **Triceps:** For the measurement the participant is sitting, probe is placed longitudinally, pointing towards the head.

III. **Abdominal:** For the measurement the participant is lying on the back, pillow under head and knee, probe is placed longitudinally, pointing towards the head. Participant is instructed to exhale slowly and as long as they can. The muscle belly of the rectus femoris must be visible.

IV. **Subscapular:** For the measurement the participant is sitting, feet on the ground. Longitudinal measure (prope pointing towards the left shoulder), prope directly on the marked area.

V. **Supraspinal:** For the measurement the participant is lying on the back, pillow under head and knee, right hand placed under the left shoulder. Probe is placed directly on the marked area (probe pointing towards the head).

VI. **Medial calf**: For the measurement the participant is lying on the back, pillow under head and knee. Longitudinal measure (probe pointing towards the head).

VII. **Multifidi:** Point at facet joints of L4/L5 (starting from the sacrum), highest point of the facet joint (the minimal distance of facet joint to the skin). For the measurement the participant is sitting, arms propped up on the thighs. Longitudinal measure (probe pointing towards the head). US settings for the multifidi are different to the general settings for all other sites: i.e., 5Mhz, Focus 31-39mm,

Depth 70mm, dyn. range 78dB, gain 92%, no spacer, no pressure.

4. Details on Training for Clinical Assessments

Assessors were trained for the bioimpedance analysis, circumference, and ultrasound measurements by an expert in anthropometry and a general practitioner with certification in sonography. In addition, Intraclass Correlation Coefficient (ICC) type 3,1 (single measure, two-way-mixed with absolute agreement) was performed to evaluate if the raters' measurements were consistent and reliable across the subjects.

Supplementary file 8: Food diary

The food diary was handed out in printed version and contained general information and tips on how to assess all food, snacks, and beverages for 3 days. The participants were instructed to choose two weekdays and 1 weekend day which were representative for their normal eating behavior (e.g. no birthday party, wedding, or public holiday).

For each day, two blank sheets are provided containing columns for time, location (at home, in a restaurant), quantity (grams, deciliters etc.), type of food and beverages (exact name, brand name etc.) and if needed a column for not eaten or drunk leftovers (grams, deciliters etc.). An example of a blank food diary is available on page 21.

Further they receive the following instructions:

1. **Instruction**

- Please write down **all food and drinks** you eat during the day and night for **3 days**, 2 of which are weekly or working days and 1 of which is a weekend day.
- For each new day, please start a new protocol sheet with the date. The attached sample protocol will help you to fill it in correctly.
- **Always carry the log with you so** you can immediately note down all the food and drinks you eat during the day, and nothing is forgotten.
- You can never write too much!!
- At the end, upload photos of the food diary via WeTransfer, instructions can be found on the last page.

2. **Please note:**

   - Just by writing down the food, your eating behavior may have changed compared to your normal everyday life. Therefore, the request: **Eat as before during the protocol days. Please do not intentionally omit or add anything you have not eaten or drunk. This is the** only way we can accurately calculate your ingested nutrients.

3. **How exactly do I have to note down the quantities?**

   - Wherever possible, please weigh food quantities with a standard kitchen scale and measure drinks as accurately as possible, e.g. with a measuring cup. Please also weigh what is left on the plate or in the glass so that only food and drinks that have actually been eaten/drunk are recorded. **Do not weigh the plate, glass, cup, etc. as well!** If it is not possible to weigh the food, please give as much information as possible using household measurements such as tablespoon, coffee spoon, coffee cup, handful, number of pieces, etc.
   - Describe each product **as accurately as possible**. For example, note the fat content and the exact name of the product as described in the attached sample protocol and the table below.

4. ***How do I record home-cooked food?***

   - For dishes you have prepared yourself, please note the quantities of **all** ingredients used in the recipe (including cooking fat, oil, cream, etc.). Since most recipes are written for several people, please weigh and write down

how much of the dish you ate (e.g. 155g of the type of vegetables, 220g of potatoes, 125g of breaded cutlet, 52g of sauce).

5. ***On the road, with acquaintances and in the canteen***
    - During the 3 days of the food record, it is advantageous in terms of accuracy to eat food that you have prepared and weighed yourself. If eating out (restaurant, canteen, invitation to a friend's house) cannot be avoided, it is sufficient to describe the menu components as precisely as possible and to estimate the portion sizes of the individual components (meat, sauce, vegetables, side dish) (e.g. in handfuls, tablespoons, portion of the plate and plate size).

6. ***Drinks***
    - Please remember to also note down all the drinks you consume. This is especially important for drinks containing calories (milk, beer, wine, lemonade, cola, etc.). For mineral water, it is important to note the brand, as this drink can contribute well to your mineral supply. It is also important to describe the drink exactly, e.g. whether it is cola or cola zero/light.

7. **How to describe food as accurately as possible**
    - Ready meals: Exact designation, manufacturer
    - Eating out: e.g. canteen, restaurant, invitations. Estimate quantity and method of preparation in household measures (tablespoon, coffee spoon, coffee cup, handful, number of pieces).
    - Milk: Type, e.g. drinking milk, buttermilk, powdered milk

- Milk substitutes: Oat, soy and almond milk, fat content, e.g. 1.5% or 3.5% fat
- Coffee cream: sweetened, unsweetened, fat content, e.g. 15%
- Yoghurt, kefir, soured milk: natural or with fruit preparation Sweetened with sugar or sweetener, fat content in %
- Cheese, curd: Variety, e.g. Edam, Camembert, cream cheese; low-fat quark, cream quark
- Eggs: Preparation method (for scrambled or fried eggs etc. with type of cooking fat)
- Spreadable fat, frying oil, salad oil: Type of fat spread: e.g. butter, margarine, normal or semi-fat, diet margarine Type of oil: e.g. olive, safflower, rapeseed, sunflower, vegetable oil etc.
- Meat, Poultry: Animal species: e.g. beef, pork, chicken, lamb, horse, roe deer Type: e.g. fillet, escalope, minced meat etc.
- Fish: Variety: e.g. redfish, saithe, herring, salmon. Method of preparation: steamed, breaded, fried (with type of cooking fat)
- Sausage, ham: Variety, e.g. Lyoner, Salsiz, Salami, Bündnerfleisch, Ham: cooked, raw, with or without fat edge
- Soups: Variety, e.g. pea soup with bacon homemade (please specify ingredients) or ready-made (product name)
- Sauces: Please specify quantity in grams Pre-pack, homemade enriched with cream, butter, oil etc.
- Bread: Type, e.g. bread rolls, wheat, rye, rye bread, whole wheat bread, sunflower seed bread
- Cake, pie, pastry: Variety, ingredients or finished product (product name)

- Sweets: Type, e.g. ice cream, chocolate bar, jelly bears, pudding, chocolate mousse; homemade or product
- Jam, honey, Nutella: normal or reduced sugar
- Potatoes: Preparation: e.g. fried, boiled potatoes, mashed potatoes (with milk, cream, incl. fat content, butter).
- Vegetables, salad: Variety, e.g. tomatoes, endive, fennel. Preparation (do not forget sauces and salad dressings)
- Fruits: Variety; with or without skin or stone, indicate ingredients when preparing (e.g. fruit salad)
- Warm drinks: Type, e.g. coffee, black tea, green tea, fruit tea, herbal tea, Ovaltine, etc. with milk or coffee cream (fat content), sweetened with sugar or sweetener
- Soft drinks: Fruit juice (pure or diluted), cola, lemonade, energy drinks, with sugar or sugar-free, etc.
- Alcoholic beverages: Wine, pure or diluted beer, straight or panache, (pilsner, wheat, Schwatzbier), schnapps, liqueur
- Water: Tap water, mineral water (product name)
- Soft drinks: Fruit juice (pure or diluted), coke, lemonade, energy drinks, with sugar or sugar-free, etc.

8. Examples of household mass if weighing is exceptionally not possible

- Bread: 1 slice
- Grease, butter: 1 knife-edge
- Cheese: 1 slice
- Processed meat, (cold cuts): 1 slice

- Sweet spreads: 1 tablespoon
- Water: 1 glass/cup
- Dessert / Yoghurt: 1 slice, bowl
- Soup: 1 plate
- Cooked potatoe: 1 piece
- Fruit e.g. apple: 1 piece (small fruits = 2 pieces)
- Sauce: 1 ladle
- Sugar: 1 sugar cube
- Milk in the coffee: 1 dash

9. Last but not least

- You will be amazed at how much food you eat every day. Taking notes is a bit tedious at first, but with time you will get into a routine.

Eating protocol day 1

Study_ID:

Day: …………… **Date:** …………… ☐ **workday** ☐ **work-free day**

1	2	3	4	5
**Time**	**Location** (at home, cafeteria, restaurant)	**Ready-to-eat Quantity (in grams, liters, deciliters, milliliters, etc.)**	**Type of food and drink (exact name, brand name if applicable, fat content, individual ingredients of dishes, etc.)**	**Remaining quantity not eaten/drunk/waste**

References

1. David Butler, Moseley L. *Schmerzen verstehen*. Springer; 2015.
2. van Wilgen CP, Nijs J. *Pijneducatie - een praktische handleiding voor (para)medici*. Bohn Stafleu van Loghum Houten; 2010.
3. Moseley GL. Joining Forces – Combining Cognition-Targeted Motor Control Training with Group or Individual Pain Physiology Education: A Successful Treatment For Chronic Low Back Pain. *The Journal of Manual & Manipulative Therapy*. 2003;11:88-94.
4. Moseley L. Combined physiotherapy and education is efficacious for chronic low back pain. *Australian Journal of Physiotherapy*. 2002;48(4):297-302. doi:10.1016/s0004-9514(14)60169-0
5. Malfliet A, Kregel J, Meeus M, et al. Applying contemporary neuroscience in exercise interventions for chronic spinal pain: treatment protocol. *Braz J Phys Ther*. Sep-Oct 2017;21(5):378-387. doi:10.1016/j.bjpt.2017.06.019
6. Lee H, Wiggers J, Kamper SJ, et al. Mechanism evaluation of a lifestyle intervention for patients with musculoskeletal pain who are overweight or obese: protocol for a causal mediation analysis. *BMJ Open*. Jul 3 2017;7(6):e014652. doi:10.1136/bmjopen-2016-014652
7. Michie S, Richardson M, Johnston M, et al. The behavior change technique taxonomy (v1) of 93 hierarchically clustered techniques: building an international consensus for the reporting of behavior change interventions. *Ann Behav Med*. Aug 2013;46(1):81-95. doi:10.1007/s12160-013-9486-6
8. Norton KI. Standards for Anthropometry Assessment. *Kinanthropometry and Exercise Physiology*. 2018:68-137.
9. Dehghan M, Merchant AT. Is bioelectrical impedance accurate for use in large epidemiological studies? *Nutr J*. Sep 9 2008;7:26. doi:10.1186/1475-2891-7-26
